# Evaluation and Future Projection of Extreme Climate Events in the Yellow River Basin and Yangtze River Basin in China Using Ensembled CMIP5 Models Data

**DOI:** 10.3390/ijerph18116029

**Published:** 2021-06-03

**Authors:** Zigeng Niu, Lan Feng, Xinxin Chen, Xiuping Yi

**Affiliations:** Hubei Key Laboratory of Critical Zone Evolution, School of Geography and Information Engineering, China University of Geosciences, Wuhan 430074, China; nzg@cug.edu.cn (Z.N.); Esthercxx961013@cug.edu.cn (X.C.); yixiuping@cug.edu.cn (X.Y.)

**Keywords:** extreme climate events, Yellow River Basin, Yangtze River Basin, CMIP5, Projections

## Abstract

The Yellow River Basin (YLRB) and Yangtze River Basin (YZRB) are heavily populated, important grain-producing areas in China, and they are sensitive to climate change. In order to study the temporal and spatial distribution of extreme climate events in the two river basins, seven extreme temperature indices and seven extreme precipitation indices were projected for the periods of 2010–2039, 2040–2069, and 2070–2099 using data from 16 Coupled Model Intercomparison Project Phase 5 (CMIP5) models, and the delta change and reliability ensemble averaging (REA) methods were applied to obtain more robust ensemble values. First, the present evaluation indicated that the simulations satisfactorily reproduced the spatial distribution of temperature extremes, and the spatial distribution of precipitation extremes was generally suitably captured. Next, the REA values were adopted to conduct projections under different representative concentration pathway (RCP) scenarios (i.e., RCP4.5, and RCP8.5) in the 21st century. Warming extremes were projected to increase while cold events were projected to decrease, particularly on the eastern Tibetan Plateau, the Loess Plateau, and the lower reaches of the YZRB. In addition, the number of wet days (CWD) was projected to decrease in most regions of the two basins, but the highest five-day precipitation (Rx5day) and precipitation intensity (SDII) index values were projected to increase in the YZRB. The number of consecutive dry days (CDD) was projected to decrease in the northern and western regions of the two basins. Specifically, the warming trends in the two basins were correlated with altitude and atmospheric circulation patterns, and the wetting trends were related to the atmospheric water vapor content increases in summer and the strength of external radiative forcing. Notably, the magnitude of the changes in the extreme climate events was projected to increase with increasing warming targets, especially under the RCP8.5 scenario.

## 1. Introduction

Global warming affects the frequency, intensity, and duration of extreme climate events such as droughts, heat waves, floods, hurricanes, and extreme cold and hot days [1,2,3]. Compared to the changes in conventional climate parameters such as the mean temperature and precipitation, extreme climate events can impose more significant stresses on human society and natural systems and can also exert severe socioeconomic and ecological impacts [4,5,6]. For example, concurrent drought and heat extremes can cause substantial decreases in barley yields worldwide, while extreme rainfall can cause floods and damage to urban infrastructure [7,8,9]. Vegetation sensitive to temperature and precipitation changes can be destroyed by extreme climate events, causing land desertification, soil erosion, and crop reduction [10,11,12,13]. Hence, it is necessary to study the spatial and temporal distribution characteristics, future development trends, and influencing factors of extreme climate events.

There are many ways to define extreme climate events, and one of them involves the indices developed by the Expert Team on Climate Change Detection and Indices (ETCCDI) [14]. This set of indices contains 16 extreme temperature indices and 11 extreme precipitation indices which are widely applied in studies of extreme climate changes and in the establishment of different climate models [15,16]. In addition, the Coupled Model Intercomparison Project (CMIP) provides a set of coordinated global climate model experiments to simulate present and future climate changes [17]. CMIP data can also be adopted in the research of extreme climate events, including the calculation of ETCCDI indices [18,19]. Compared to CMIP Phase 3 (CMIP3) data, the performance of CMIP Phase 5 (CMIP5) data in the simulation of extreme climate indices exhibits certain improvements on the global and regional scales [20,21,22].

On the global scale, increased warm events and decreased cold events have been reported over the past few decades, which indicates that changes are generally expected in a warming world [23,24,25]; extreme precipitation events will also increase in a warming climate due to the increased atmospheric humidity [26]. Extreme climate events in different regions worldwide always reveal different trends because of the contributions of atmospheric dynamics and thermodynamics [26,27]. For example, extreme precipitation exhibits a notable positive relation with a warming climate in midlatitude locations but a weak or negative relation in the tropics [26]. This result can also be observed in the northeastern and southeastern U.S. [28,29] and Southeast Asia [30,31].

The extreme climate events in China are greatly affected by monsoons and geographical environments, such as territory and terrain conditions [32]. In addition, the urbanization in China has rapidly increased with economic development since the 1970s, and this increase has led to local climate changes [33]. Large-scale atmospheric circulation parameters also cause notable impacts on the extreme climate events in China, especially the El Niño-Southern Oscillation (ENSO), the Atlantic Multidecadal Oscillation (AMO), and the Pacific Decadal Oscillation (PDO) [34,35,36].

Many studies have noted that significant increases have occurred in the frequency of warm extremes and decreases in cold extremes in China over the past few decades. For example, Shi et al. [37] mentioned the cold spell duration indicator (CSDI) decreased and the warm spell duration indicator (WSDI) increased in almost all parts of China during 1961–2005; Wu et al. 2019 [38] found that there was a significant increase in the frequency of compound dry/warm and wet/warm extremes while there was a decrease in compound dry/cold and wet/cold extremes for the period of 1988–2014 relative to 1961–1987 in China, which are consistent with global warming [39,40]. However, increase trends of extreme rainfall events coupled with decreased dry spells have been observed in many regions in China over the past century, such as the monsoon regions in China during 1964–2014 [41], northwestern China (1961–2010), and southeastern China (1961–2016) [42,43]. In northeastern and southwestern China, a notable drying tendency has been confirmed [34,42]. In the future, model calculations suggest that additional warming might increase extreme events in some areas, although there are considerable uncertainties in predicting future climates in specific localized areas. For example, an additional half a degree caused by global warming may increase the magnitude of extreme precipitation events [44]. However, Wang et al. [45] suggested a negative scaling of extreme precipitation with very high temperatures, thus raising doubts about future increases in precipitation extremes.

These prior studies have mainly focused on China as a whole and revealed large-scale climate changes, but regional-scale research may help us to better understand the characteristics of climate change in different areas of China due to its different locations and terrains [16,46]. Furthermore, the impacts of extreme climate events on regions with different land use types are also different [41,47,48,49]. The Yangtze River Basin (YZRB) and Yellow River Basin (YLRB) are important population settlements and water supply sources, and they host several important economic belts [50]. In addition, the ecological environment in these river basins is fragile and more susceptible to extreme climate events [51,52]. Studies have focused on extreme climate events in the YZRB and YLRB, mainly employing station-observed data to study the spatiotemporal distribution characteristics of short-term extreme climate indices [53,54,55,56,57,58]. However, there is a lack of research on continuous spatial scales, as well as on the trends of extreme climate events in different future periods. Moreover, the impacts of atmospheric circulation patterns on extreme climate events also need to be further analyzed.

In this study, we focused on the spatiotemporal distributions of extreme temperature and precipitation events in the YLRB and YZRB during the historical periods and different scenarios in the 21st century. We simulated several extreme climate indices based on data from multiple CMIP5 models in the YZRB and YLRB and compared the performance with observed data. Finally, we examined the influencing factors on the changes in extreme climate events in these two basins.

## 2. Materials and Methods

### 2.1. Study Area and Observed Data

**Figure 1:**
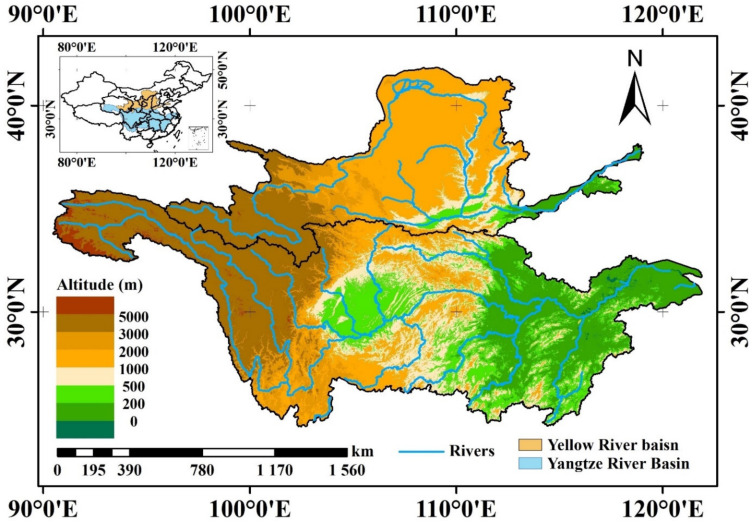
Geographical information of the Yellow River basin and Yangtze River basin.

The Yangtze River and Yellow River are the first and second longest rivers, respectively, in China, and their basin areas total approximately 2.55 × 10^6^ km^2^, which accounts for nearly 26.6% of the landmass of China (Figure 1). The YZRB and YLRB consist of multiple economies, and more than 500 million people live in these basins, accounting for approximately 40% of the Chinese population [54,56]. The Yangtze River and Yellow River both originate on the Qinghai-Tibet Plateau at elevations exceeding 5000 m above sea level but ultimately flow into the East China Sea and Bohai Sea, respectively. Because of the sufficient water supply, the YZRB and YLRB are important wheat and maize production areas in China [52,59], but the complex terrain and climatic conditions threaten the ecological environment of these two river basins.

The observed daily mean air temperature (Tm), daily maximum temperature (Tx), daily minimum temperature (Tn), and daily precipitation (Pre) over the period of 1961–2005 were obtained from 2472 national meteorological stations (excluding the two offshore island stations of Xisha and Coral Island) of the National Meteorological Information Center (NMIC), China Meteorological Administration (CMA; http://data.cma.cn/, accessed on 24 September /2019). The station construction and observation methods are consistent with the standards issued by the World Meteorological Organization (WMO), and the data have undergone strict quality control measures to ensure that the accuracy of the daily weather datasets approaches is 100% [60,61,62]. Hence, the data were converted to grid data at a 0.5° × 0.5° spatial resolution using the thin plate spline (TPS) method. There were a few missing values in the observational data, such as the precipitation data in 2002 and, therefore, the reference period was set to 1971–2000.

### 2.2. CMIP5 Model Simulations

The daily Tm, Tx, and Tn of 14 CMIP5 models and Pre of 10 CMIP5 models were adopted in this study and are available from the Earth System Grid Federation (ESGF, https://esgf-node.llnl.gov/projects/cmip5/, accessed on 2 October 2020) repositories. Table 1 provides the primary information of the various CMIP5 models with different horizontal and atmospheric resolutions. The data included historical simulations (from the 19th century to 2005) and future projections (2006–2300). The spatial distribution of future climate changes over the 30-year periods of the 2020s (2010–2039, or beginning-of-century), 2050s (2040–2069, or mid-century), and 2080s (2070–2099, or end-of-century) were analyzed relative to the reference period. Two different representative concentration pathways (RCP4.5 and RCP8.5) of future emissions were chosen, and they were named based on the radiative forcing in 2100, i.e., at 4.5 and 8.5 W/m^2^, respectively [63,64]. All models were bilinearly interpolated to a common 0.5° × 0.5° grid, consistent with the observations.

**Figure 2:**
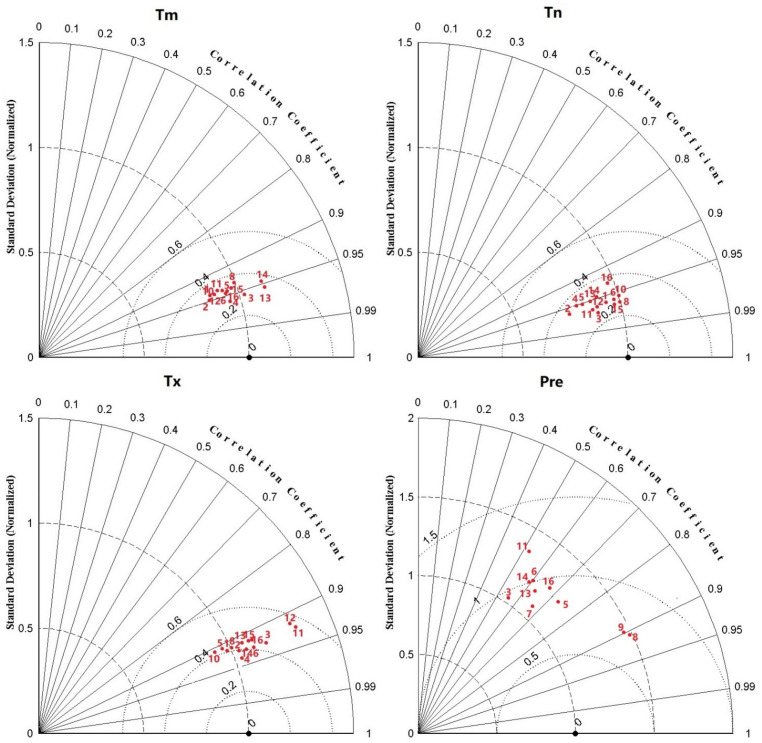
Taylor diagrams for the simulations of 30-annual mean Tm, Tx, Tn, and Pre of the two basins during the 30-year period (1971–2000).

Consequently, we generated Taylor diagrams to visualize the model simulation performance of the 30-annual mean values in the two basins relative to the observations of Tm, Tx, Tn, and Pre in the historical period (Figure 2) [65]. We selected 10 models for the estimation of Pre (CCSM4, CMCC-CMS, CSIRO-Mk3.6.0, CanESM2, HadGEM2-AO, HadGEM2-CC, IPSL-CM5B-LR, MPI-ESM-LR, MPI-ESM-MR, and INMCM4), as well as 14 models for the estimation of Tm, Tx, and Tn (ACCESS1.0, ACCESS1.3, CCSM4, CMCC-CM, CMCC-CMS, CSIRO-Mk3.6.0, HadGEM2-AO, HadGEM2-ES, IPSL-CM5A-MR, IPSL-CM5B-LR, MPI-ESM-LR, MPI-ESM-MR, NorESM1-M, and INMCM4).

**Figure 3:**
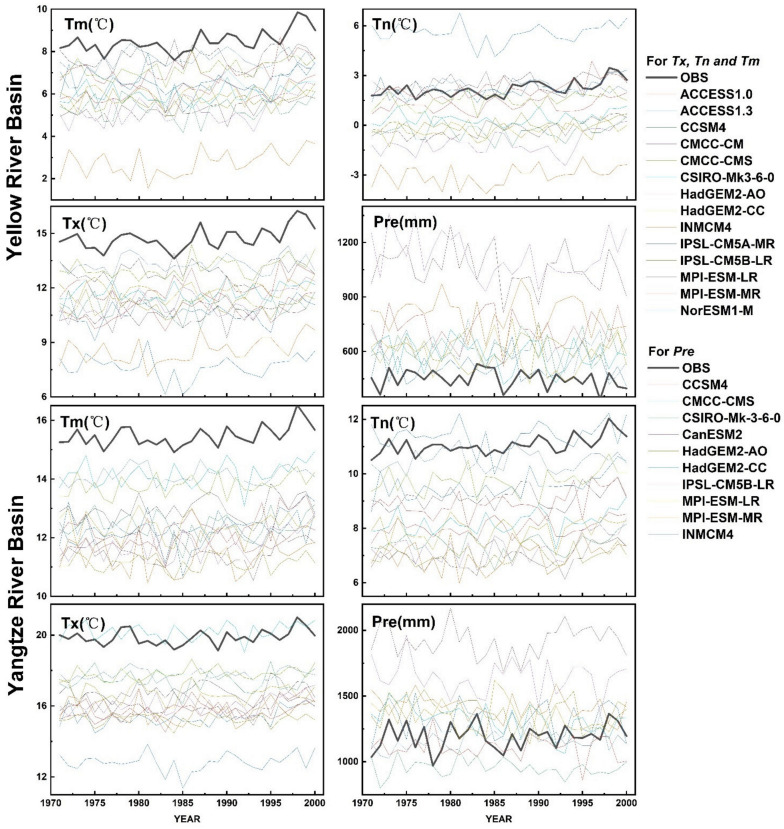
Annual mean Tm, Tn, Tx, and Pre from observed data and original CMIP5 model data during 1971–2000 in the Yellow River Basin and Yangtze River Basin.

Here, we provide mean and trends of the Tm, Tn, Tx, and Pre values during 1971–2000 for the YLRB and YZRB in Table 2 and Figure 3 to compare the difference between the original CMIP5 data and observed data. The multi-year mean Tm, Tn, Tx, and Pre were 8.49, 2.22, 14.75 °C, and 499.18 mm in the YLRB, and the intervals of model values were 2.76–7.71 °C, −3.04–5.56 °C, 7.65–13.31 °C, and 515.1–1124.85 mm for Tm, Tn, Tx, and Pre, respectively. In the YZRB, the observed Tm, Tn, Tx, and Pre were 5.47, 11.06, and 19.88 °C and 1192.65 mm, and the intervals of the model values were 11.15–14.18, 6.94–11.39, 12.83–20.22, and 954.07–1916.59 mm for Tm, Tn, Tx, and Pre, respectively. In addition, the observed Tm, Tn, and Tx increased significantly at the rate of 0.34, 0.33, and 0.36 °C/decade in the YLRB during 1971–2000, while the Pre did not decrease significantly at −12.38 mm/decade. In the YZRB, the observed Tm and Tn increased significantly at 0.19 and 0.23 °C/decade during 1971–2000, while the increase trends of Tx and Pre were not significant (0.14 °C/decade and 26.14 mm/decade, respectively). At the same time, we also noticed that there was no single CMIP5 model which could better capture the annual mean value and multi-year trends of the observed temperature and precipitation data from 1971 to 2000. For example, in the YLRB and YZRB, the models usually underestimated the Tm, Tn, and Tx but overestimated the Pre, while the trends of MPI-ESM-MR model were close to the observed values in YLRB. In the YZRB, the INMCM4 model could better capture the multi-year mean Pre, but no model could better capture the trends of the observed Tm, Tn, and Tx.

### 2.3. Climate Extreme Indices

To reflect the extreme temperature and precipitation in multiple aspects, 14 extreme climate indices recommended by the ETCCDI were employed in this study (Table 3), including seven extreme temperature indices (the diurnal temperature range (DTR), the numbers of summer days (SU), the number of ice days (ID), the highest daily maximum temperature (TXx), the lowest daily minimum temperature (TNn), the warm spell duration index (WSDI), and the cold spell duration index (CSDI)) and seven extreme precipitation indices (the highest 5-day precipitation (Rx5day), the extremely wet-day precipitation (R99pTOT), the heavy precipitation days (R20mm), the total wet-day precipitation (PRCPTOT), the precipitation intensity (SDII), the consecutive dry days (CDD), and the consecutive wet days (CWD)) [20,21,40,66,67,68,69,70].

The TXx, TNn, and DTR indices indicated the intensity of the extreme temperature, while the SU and ID indices represented the frequency and intensity, respectively, of extreme-temperature events, and the WSDI and CSDI indices represented the duration of extreme-temperature events. The Rx5day, R99pTOT, R20mm, PRCPTOT, and SDII indices represented different ways to assess extreme precipitation, and the CDD and CWD indices helped to distinguish between dry and humid areas in the two basins. The Rx5day and CDD indices can also be applied to evaluate potential floods because persistent heavy rainfall promotes the occurrence of floods and subsequent landslides [71]. These indices can also be classified into four categories: absolute indices, threshold indices, percentile indices, and duration indices [69,72]. The nonparametric Mann-Kendall trend test is applied to establish whether the trends of these indices are significant [73].

### 2.4. CMIP5 Data Processing

#### 2.4.1. Delta Change Method

The coarse resolution of the above models cannot provide reliable information at the local and regional scales, and a single CMIP5 model data cannot capture the multi-year average value and trends of the observed data. Thus, it is necessary to compensate for this deficiency by the application of downscaling methods, such as dynamical downscaling and statistical downscaling [69,74,75,76]. Dynamical downscaling produces finer-scale global climate models (GCMs) by nesting fine-resolution regional climate models (RCMs) [77,78], while statistical downscaling establishes and applies the historical statistical relationships between large-scale atmospheric variables and local climate variables [74,79]. In this study, we applied statistical downscaling because of its higher computational efficiency.

The delta change method is a simple statistical downscaling method and is applied to correct the bias of the simulated temperature and precipitation data. The equation is given as follows:

(1) $$x_{\mathrm{cor},i,j,k}{=x}_{\mathrm{sim},i,j,k}{+(\bar{x}}_{\mathrm{obs},i,j,k}{-\bar{x}}_{\mathrm{sim},i,j,k})$$

where $x_{\mathrm{sim},i,j,k}$ and $x_{\mathrm{cor},i,j,k}$ are the simulated and bias-corrected i-th meteorological variables, respectively, at the j-th grid point on the k-th day, and ${\bar{x}}_{\mathrm{sim},i,j,k}$ and ${\bar{x}}_{\mathrm{obs},i,j,k}$ are the 30-year (1971–2000) averages of the simulated and observed i-th meteorological variables, respectively, at the j-th grid point on the k-th day [70,79,80].

#### 2.4.2. Reliability Ensemble Averaging Method

Previous studies noted that individual models perform differently in the simulation of different extreme indices [20,40,67,80,81,82,83]. Moreover, the performance of a multi-model ensemble is superior to that of most individual models [21,67,84]. In this study, we adopted the reliability ensemble averaging (REA) method to estimate the simulation extreme indices, and the multi-model weighted average change is defined as:

(2) $$\tilde{\Delta T}=\tilde{A}\left(\Delta T\right)=\frac{\sum_{i}R_{i}\Delta T_{i}}{\sum_{i}R_{i}}$$

where the operator $\tilde{A}$ represents the REA operation and ∆T_i_ is the simulated change in the individual model output [85]. Variable R_i_ is a weight formulated as:

(3) $$R_{i}=\left[{\left(R_{B,i}\right)}^{m}\times {\left(R_{D,i}\right)}^{n}\right]=\left\{{\left[\frac{\varepsilon_{T}}{{\mathrm{abs}(B}_{T,i})}\right]}^{m}{\left[\frac{\varepsilon_{T}}{{\mathrm{abs}(D}_{T,i})}\right]}^{n}\right\}$$

where R_B,i_ is a measure of the model performance criterion as a function of a bias factor (B_T,i_); R_D,i_ is a measure of the model convergence criterion as a function of a distance factor (D_T,i_); B_T,i_ is the bias between the simulated and observed output values over the baseline period (1971–2000); and D_T,i_ is calculated by an iterative procedure. The initial hypothesis of D_T,i_ was given by the difference between each model change and the simple ensemble averaging (defined as the mean of equally weighted models) change. Thereafter, the first guess of $\tilde{\Delta T}$ was computed with Equations (2) and (3) and then subtracted from each model change to recalculate D_T,i_. The iteration was repeated until the procedure converged. The m and n parameters were employed to weigh each criterion, while ε_T_ is the difference between the maximum and minimum 10-year moving average values of the series after linear detrending [85].

**Figure 4:**
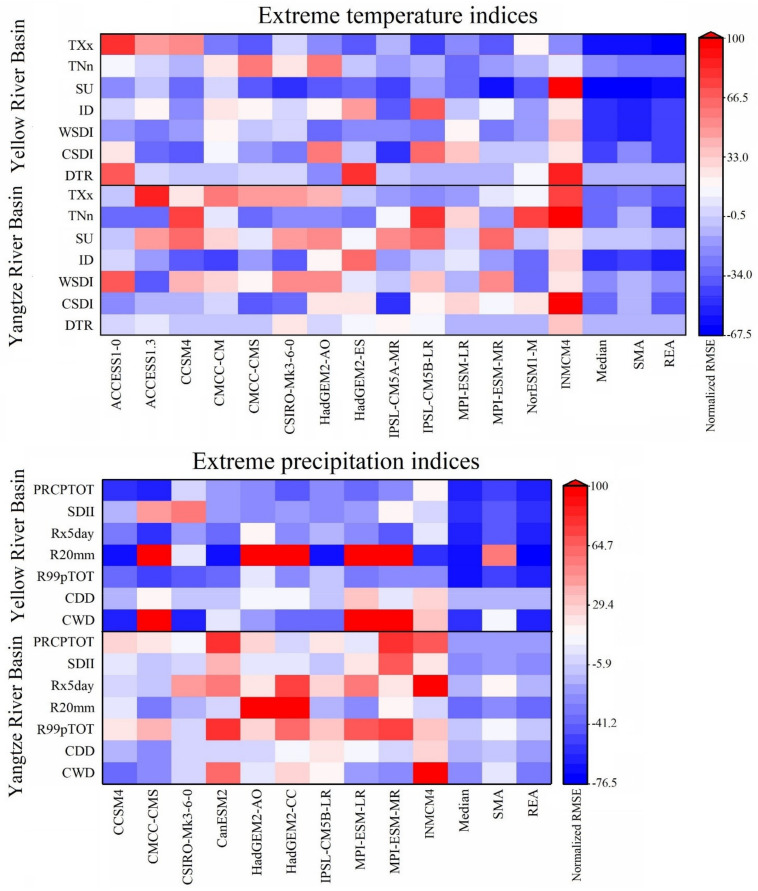
The relative RMSEs of extreme weather indices between the observed and the bias-corrected GCM simulations over the Yellow River Basin and Yangtze River Basin.

The performance profiles of the relative root mean square errors (RMSEs) of the extreme indices simulated by the CMIP5 models with respect to the observations in the 1971–2000 climatology are shown in Figure 4. To depict the RMSEs of the multiple variables at the same scale, the relative RMSE is defined as:

(4) $${\mathrm{RMSE}}_{j}^{R}=100\frac{\left({\mathrm{RMSE}}_{j}{-\mathrm{RMSE}}_{\mathrm{median}}\right)}{{\mathrm{RMSE}}_{\mathrm{median}}}$$

where RMSE_j_ is the RMSE of the j-th model and RMSE_median_ is the median RMSE of the 14 and 10 CMIP5 models for extreme temperature and precipitation indices, respectively [20,86].

Here, we applied three multi-model ensemble methods: the multi-model median (Median), simple model averaging (SMA, where each model is weighted equally), and REA methods. As shown in Figure 4, the performance of the individual models differed in terms of the simulation of the different extreme indices, especially the multi-model ensemble results. The RMSEs of the median, SMA, and REA were obviously lower than the RMSEs of single model; especially the REA results outperformed those of the individual models, and the values in the Yellow River basin were totally lower than those in the Yangtze River basin. Thus, we chose the REA results for further analysis and discussion in the following section.

To evaluate the robustness of the model’s estimation in the future period, we calculated the uncertainty credibility of multi-model collective signals (SN):

(5) SN=DN/DS 

where the DN is the absolute differences of REA values between 2006–2100 and 1971–2000. The DS can be calculated as:

(6) $$\mathrm{DS}=\sqrt{\frac{1}{N}\sum_{i=0}^{N}{\left(E_{i}-\bar{E}\right)}^{2}}$$

where N is the number of models and the E_i_ is the annual mean value of i-th model in the future period, and E is the annual mean REA value in the future. When the SN is larger than 1, the model output result is robust, and the result is uncertain if SN is less than 1 [87].

## 3. Results

### 3.1. Spatial Distribution of the Multiyear Mean Extreme Climate Indices Based on the Observed Data

#### 3.1.1. Extreme Temperature Indices

**Figure 5:**
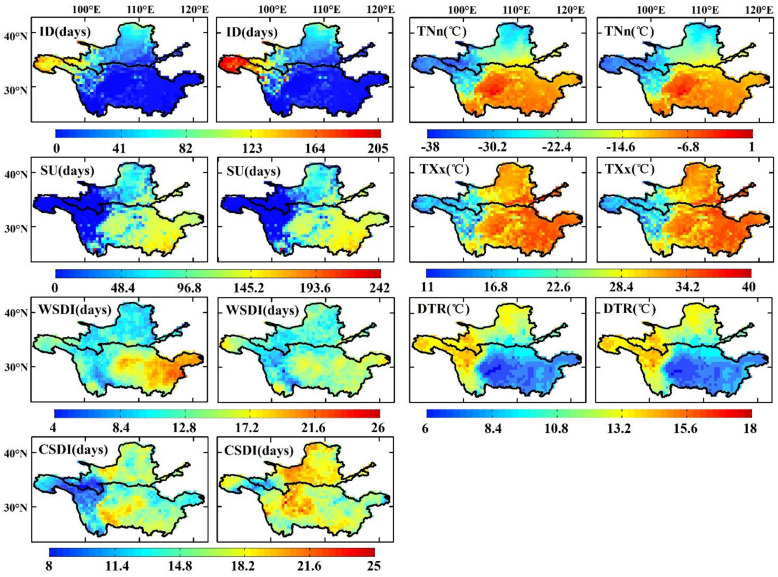
Spatial distribution of seven extreme temperature indices for the annual mean observations (OBS, the first and third columns) and simulated (REA, the second and forth columns) values in the 1971–2000 period over the Yellow River Basin and Yangtze River Basin.

The spatial distribution of the annual mean observed and simulated extreme temperature indices from 1971–2000 is shown in Figure 5. In general, the ensemble TXx, TNn, DTR, ID, and SU index values were in good agreement with the observed data in the two basins, but the modeled WSDI and CSDI indices did not agree well with the observed indices (Table 4). The regional mean observed and simulated TXx ranged from 11 to 40 °C in the YLRB and YZRB, and the TNn ranged from −23.68 to −11.62 °C. The TXx and TNn index values were generally higher in the southeastern YZRB and the Sichuan Basin, and both were lower in the western region of the two basins. The observed and simulated WSDI and CSDI values were in the range of 4–26 and 8–25 days in the two basins, and the SU and ID ranged from 0 to 205 days and 0 to 242 days in the two basins. In addition, the DTR in the YLRB and YZRB ranged from 6 to 18 °C.

The regional mean differences between the ensembled and observed TXx and TNn index values were 0.46 and 0.69 °C, respectively, in the YLRB and 0.15 and −0.15°C, respectively, in the YZRB. The TXx and TNn index values mainly revealed positive errors on the Loess Plateau and the central-western region of the two basins, and the maximum values were 1.5 and 2 °C, respectively, while negative errors mainly occurred in the western and central-eastern regions of the YZRB at −1.7 and −1.9 °C, respectively. The SU index value in the western region of the two basins was almost zero days, and the maximum value appeared in the southeastern YZRB at approximately 241 days. The average differences in the SU index values in the YLRB and YZRB were 1.15 and 2.56 days, respectively, and the maximum difference was observed in the Sichuan Basin at 10 days. The ID index value in most regions of the YZRB was smaller than 10 days, while the value was smaller than 100 days in most regions of the YLRB. A high ID index value mainly occurred in the western region (generally larger than 100 days), and the maximum value was approximately 202 days. Finally, the maximum and minimum differences of ID primarily occurred in the western and central regions of the two basins (10 and −7 days, respectively).

The WSDI index value in the YLRB and western YZRB was generally lower than that in the eastern YZRB, with maximum and minimum values of approximately 6 and 26 days, respectively. However, the spatial distribution of the difference was the opposite to that of the observed values in general: the maximum and minimum differences occurred in the YLRB and eastern YZRB, and the difference ranged from −13 to 8 days. In addition, low CSDI index values were observed in the western regions of the two basins, with a minimum value of 8 days, and the highest value occurred in the Sichuan Basin at 22 days. The maximum and minimum differences in the CSDI index were observed in the western region of the two basins and the Sichuan Basin (−7 and 13 days, respectively), with absolute REs of 20.75 and 16.51% in the YLRB and YZRB, respectively. Finally, the DTR index values in the western regions of the two basins and on the Loess Plateau were much higher than those in the Sichuan Basin and the middle and lower reaches of the YZRB, and the value ranged from 6–17 °C. The ensemble DTR index values were higher than the observed values in the Sichuan Basin, with a maximum value of 0.11 °C, but were lower in the central region of the two basins, with a minimum value of −0.03 °C.

#### 3.1.2. Extreme Precipitation Indices

**Figure 6:**
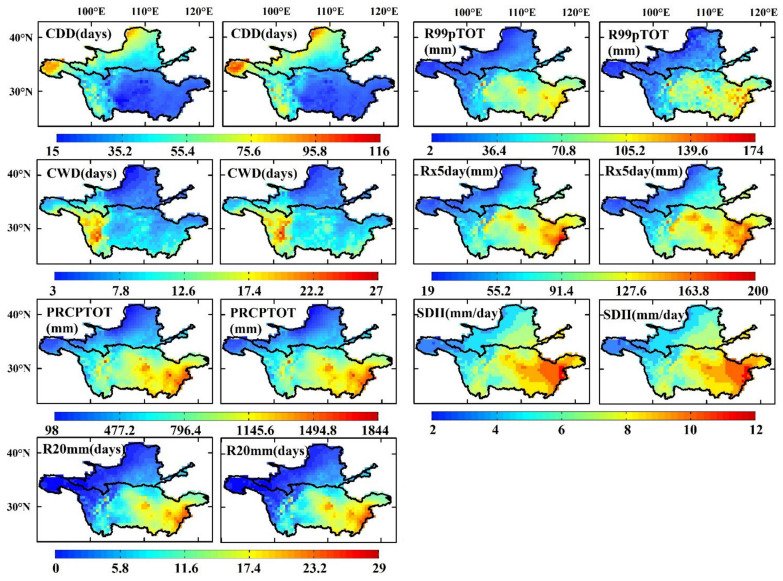
Same as in Figure 5 but for the extreme precipitation indices.

Figure 6 shows the spatial patterns of the observed and simulated extreme precipitation indices in the YLRB and YZRB. In general, the ensemble values agreed very well with the observed values, especially the PRCPTOT index, but the ensemble Rx5day, CDD, and CWD index values were always larger than the observed values. Except for the CDD and CWD indices, all the other observed extreme precipitation indices were high in the central-eastern YZRB and low in the northwestern YLRB. The highest values of the PRCPTOT, Rx5day, R20mm, SDII, and R99pTOT indices were 1842 mm, 200 mm, 28 days, 12 mm/day, and 162 mm, respectively, and the minimum values were 106 mm, 19 mm, 0 days, 2 mm/day, and 6 mm, respectively. The maximum values of the CDD and CWD indices were observed in the northwestern YLRB and western YZRB (109 and 26 days, respectively), and the minimum values occurred in the Sichuan Basin and northern Loess Plateau (15 and 3 days, respectively).

The regional mean observed and ensemble PRCPTOT were 411.24 and 438.41 mm in the YLRB, and 963.14 and 959.81 mm in the YZRB; the observed and ensemble SDII were 5.85 and 5.90 in the YLRB and 7.78 and 7.85 in the YZRB (Table 4). The spatial distributions of the differences in the PRCPTOT and SDII index values were scattered, and their extreme value distributions were not distinct, which was also true for those of the R99pTOT index. The differences in the R20mm and Rx5day index values were generally large in the central YZRB, with maxima of 33 mm and 1.7 days, respectively, but minimum values were observed in the southern YLRB and eastern YZRB (−0.6 days and −21 mm, respectively). Finally, the ensemble CDD and CWD index values were overestimated in the two basins, and the regional mean differences were 3.39 and 0.63 days, respectively, in the YLRB and 3 and 0.83 days, respectively, in the YZRB. The maximum difference in the CDD index value occurred in the western regions of the two basins at 20 days, and the CWD index exhibited the minimum difference at −3 days (Table 4 and Figure 6).

### 3.2. Future Changes in the Extreme Climate Indices under the RCP4.5 and RCP8.5 Scenarios

#### 3.2.1. Extreme-Temperature Events

**Figure 7:**
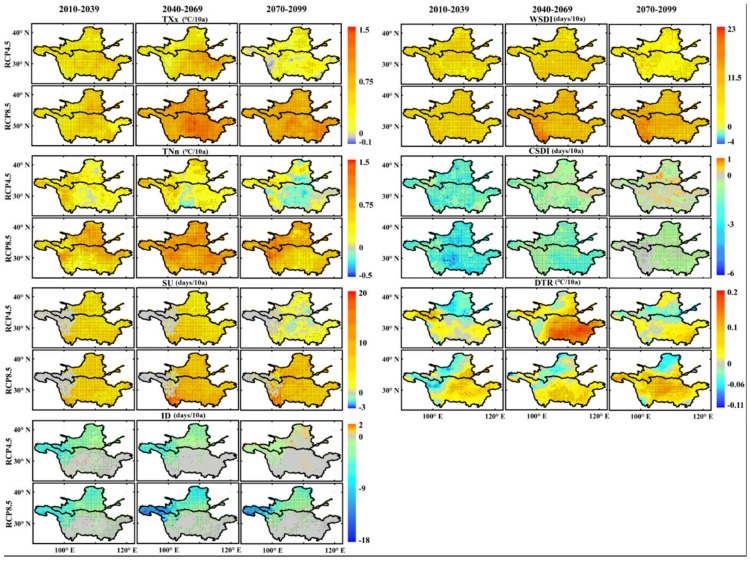
Spatial distributions of trends in seven extreme temperature indices for the 2020s (2010–2039), 2050s (2040–2069), and 2080s (2070–2099) periods in the Yellow River Basin and Yangtze River Basin under the RCP4.5 and 8.5 scenarios. The black solid dots indicate the trends are significant at the 95% significance level.

Figure 7 shows the spatial distribution of the changes in the extreme temperature indices over the three periods of the 21st century based on the RCP4.5 and RCP8.5 scenarios. Under the RCP4.5 scenario, the TXx index showed an increase trend in the different periods in most regions of the YZRB and YLRB, and the largest increase occurred in the central-eastern part of the two basins in the 2050s period at approximately 0.8 °C/decade. However, in the 2080s period, a slight decline in the TXx index was found in the western YZRB, with a minimum value of approximately −0.1 °C/decade. Under the RCP8.5 scenario, the TXx index revealed the highest increase trend in the central YZRB in the 2050s period (1.5 °C/decade) and the lowest increase trend in most regions of the two basins (lower than 0.1 °C/decade). The TNn index in the 2080s period exhibited the largest decline in the central YZRB (−0.5 °C/decade) under the RCP4.5 scenario, but under the RCP8.5 scenario it showed increase trends in the three periods; the highest trend was found in the west region of the two basins (1.5 °C/decade) in the 2080s period.

The SU index showed the highest increase trend in the YZRB in the 2050s period under the RCP4.5 scenario (approximately 10 days/decade), while in the 2080s period, it decreased at a rate of −3 days/decade in the central YZRB. Under the RCP8.5 scenario, the largest increase in the SU index also occurred in the western YZRB in the 2050s period, and the regional trends of the SU index in the 2020s and 2080s periods were 4.1 and 6.1 days/decade, respectively. The ID index mainly exhibited decrease trends in the YLRB and the western regions of the two basins under the RCP4.5 and RCP8.5 scenarios. The largest decline in the ID index under the RCP4.5 scenario occurred in the western YZRB and YLRB in the 2050s period (−4.5 days/decade), while in the 2080s period, slight increases in the ID index occurred in the YLRB, at a maximum value of 2 days/decade. Under the RCP8.5 scenario, the ID index decreased in the three periods, and the largest decline was found in the western YZRB at −18 days/decade in the 2050s period.

The WSDI index under the RCP4.5 scenario revealed the largest increase in the 2050s period, but its increase trend decreased in the 2080s period, and there was a downward trend in some places (the largest decline was −4 days/decade). However, under the RCP8.5 scenario, the increase rate of the WSDI index increased over time, and the maximum rate occurred in the western YZRB in the 2080s period (23 days/decade). In addition, the CSDI index under both the RCP4.5 and RCP8.5 scenarios exhibited the largest declines in the 2020s period in the two basins, with a minimum value of approximately −6 days/decade, but slight increases occurred in the two basins in the 2050s and 2080s periods under the RCP4.5 scenario, while the maximum value was 1 day/decade. Under the RCP8.5 scenario, the regional decrease rates of the CSDI index in the two basins were −1.2 and −0.3 days/decade.

Finally, the DTR index in the YZRB generally revealed an increase trend under the RCP4.5 scenario, especially in the middle and lower reaches, and the maximum value was 0.2 °C/decade in the 2050s period. However, under the RCP8.5 scenario, the largest increase was found in the central YZRB in the 2080s period (approximately 0.15 °C/decade). The DTR index generally exhibited a decrease trend in the northwestern region of the two basins, and the largest decline occurred in the 2020s period under the RCP8.5 scenario at −0.11 °C/decade.

#### 3.2.2. Extreme Precipitation Events

**Figure 8:**
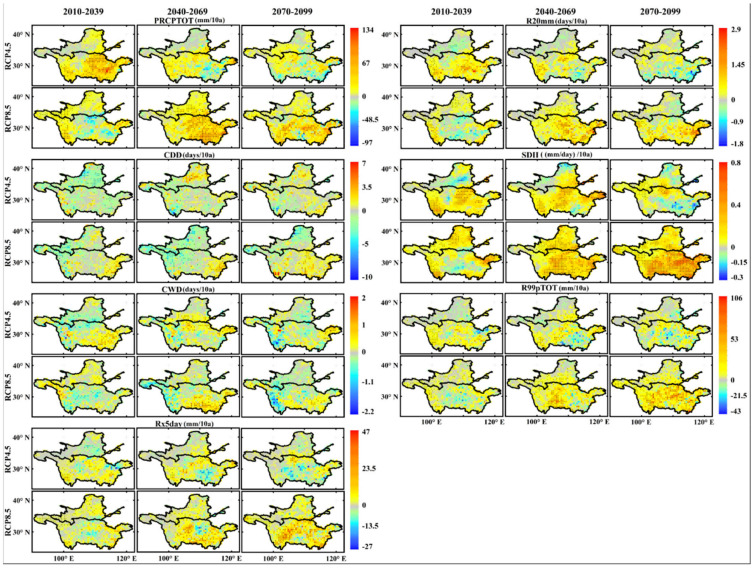
Same as in Figure 7 but for the extreme precipitation indices.

Figure 8 shows the spatial distribution of the changes in the precipitation extremes in the three periods based on the RCP4.5 and RCP8.5 scenarios. The Rx5day index in the YZRB exhibited the largest decline in the 2080s period under the RCP4.5 scenario at −28 mm/decade; under the RCP8.5 scenario, it revealed the highest increase trend in the 2080s period in the YZRB at 47 mm/decade. A regional decrease trend only occurred in the YLRB in the 2020s period at −0.37 mm/decade under the RCP4.5 scenario.

The R99pTOT index under the RCP4.5 scenario generally exhibited increase trends in the YZRB and slight increases in the YLRB in the 21st century, and the regional mean trends were 5.8, 5.5, and 3.5 mm/decade, but the largest decline also occurred in the YZRB in the 2080s period at −43 mm/decade. Under the RCP8.5 scenario, the R99pTOT index in the YLRB and YZRB revealed the highest increase trend in the two basins, especially in the 2080s period in the YZRB, with maximum and average mean values of 106 and 21 mm/decade, respectively. The PRCPTOT index exhibited the highest increase trend in the 2020s period in the YZRB at a rate of 134 mm/decade and revealed the largest decline in the 2050s period at approximately −80 mm/decade under the RCP4.5 scenario. Under the RCP8.5 scenario, the PRCPTOT index had a decrease trend in the central region of the two basins in the 2020s period, with the largest decline equaling approximately −97 mm/decade, and exhibited the highest increase trend in the 2050s period at approximately 120 mm/decade.

Under both the RCP4.5 and RCP8.5 scenarios, the SDII index values in the two basins revealed slight increases in the three periods, and the regional mean trends were all lower than 0.3 mm/day/decade. The highest increase trends (approximately 0.8 mm/day/decade) were primarily found in the eastern region of the two basins in the 2050s period under the RCP4.5 scenario. In the 2020s and 2080s periods, there were a few decreases in the central YLRB and southeastern YZRB at approximately −0.3 mm/day/decade. Under the RCP8.5 scenario, there was a slight decrease in the central YZRB in the 2020s period at approximately −0.15 mm/days/decade, while in the 2050s and 2080s periods, the increase trends in the YZRB were 0.18 and 0.27 mm/day/decade, respectively. The R20mm index also increased slightly in the three periods under the RCP4.5 scenario, and increases generally occurred in the YZRB. In the 2080s period, the R20mm index in the YZRB exhibited a decrease trend, with highest and regional mean values of −1.8 and −0.02 days/decade, respectively. Under the RCP8.5 scenario, slight decrease trends of the R20mm index were found in the central YZRB in the 2020s period at approximately −0.4 days/decade, but the regional mean trend in the two basins was 0.15 days/decade, and the largest increases appeared in the YZRB in the 2050s at 2.9 days/decade.

Finally, under the RCP4.5 scenario, the CDD index mainly revealed a decrease trend in the YLRB and YZRB in the 2020s period at rates of −1.22 and −0.42 days/decade, while in the 2050s period, the CDD index increased at a rate of 0.18 days/decade in the YLRB but decreased at a rate of −0.25 days/decade in the YZRB. In the 2080s, the CDD index values in the two basins both increased at a rate of 0.1 days/decade. Under the RCP8.5 scenario, except in the YZRB in the 2080s period (0.55 days/decade), the CDD index values all revealed decrease trends, and the minimum rate was approximately −10 days/decade. The CWD index in the two basins exhibited decrease trends in the 2020s and 2080s periods (at −0.04 and −0.02 days/decade, respectively) but increases in the 2050s period at a rate of 0.15 days/decade. Under the RCP8.5 scenario, the highest increase trend of the CWD index was observed in the southeastern YZRB in the 2050s period at 2 days/decade, and the highest decrease trend occurred in the western YZRB with −2.2 days/decade.

### 3.3. Long-Term Variations in the Extreme Climate Events in the YLRB and YZRB

**Figure 9:**
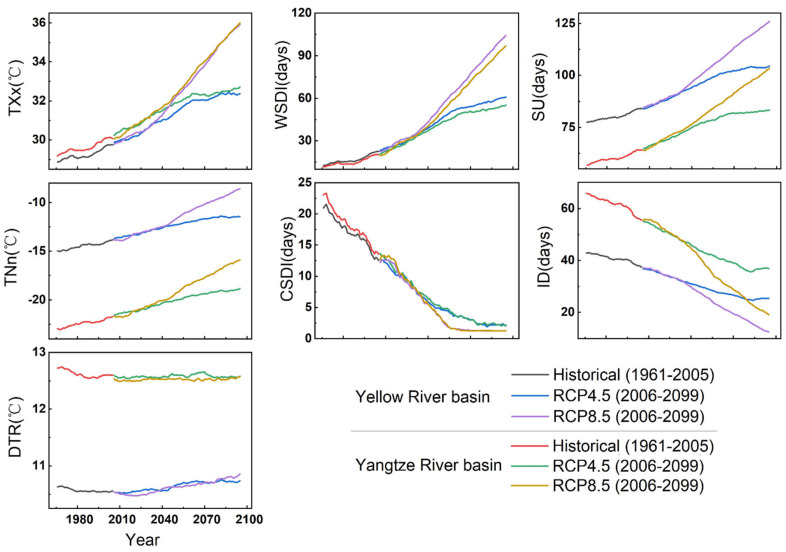
Annual regional mean of seven extreme temperature indices for the history and future periods.

**Figure 10:**
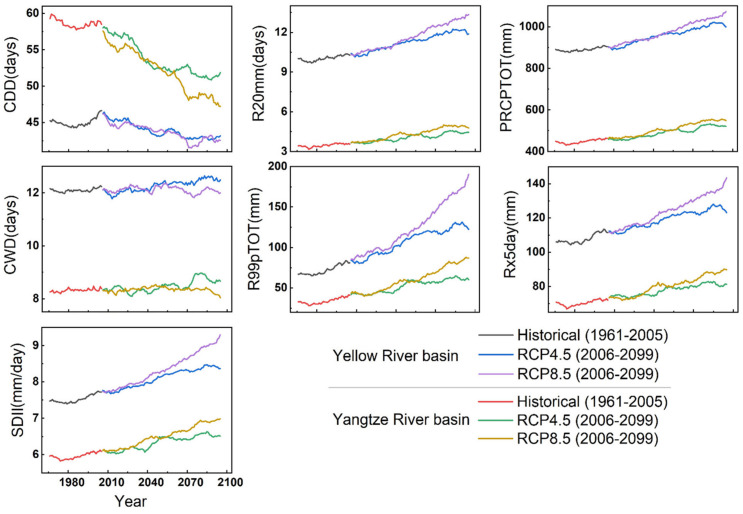
Annual regional mean of seven extreme precipitation indices for the historical and future periods.

Figure 9 and Figure 10 show the projected changes in the regional mean extreme temperature and precipitation indices under the RCP4.5 and RCP8.5 scenarios. The variations in the extreme temperature indices in the YLRB and YZRB exhibited a strong consistency between the historical and future periods. There were general increases in most extreme temperature indices but notable decreases in the CSDI and ID indices in both basins. The trends of extreme temperature indices in the early 21st century were relatively similar under the RCP4.5 and RCP8.5 scenarios, but the values rapidly increased after 2040 under the RCP8.5 scenario in the two basins, except the DTR index. The values changed less after 2060, and the ID index even revealed an increase trend after 2080 under the RCP4.5 scenario. The extreme precipitation indices revealed trends were similar to those of the extreme temperature indices in the two basins, but the values in the YZRB were of a greater magnitude of change than those in the YLRB, except for the CDD index, indicating a wetter climate in the YZRB in future decades. However, after 2080, most of the indices indicated more notable trends than those in the early period under the RCP8.5 scenario but revealed the opposite trends under the RCP4.5 scenario.

Table 5 shows the trends of extreme climate indices in the YLRB and YZRB for the historical period and under the RCP4.5 and 8.5 scenarios in the future period. Almost all the extreme climate indices showed significant trends in the two basins, except the CWD under the RCP8.5 scenario in the YLRB and YZRB. The extreme cold events in the two basins all showed down trends, like ID, CSDI, and CDD, and the decline in the YLRB was higher than that in the YZRB. For example, the trends of ID in the YLRB ranged from −2.32 to −4.95 days/decade (*p* < 0.01), while in the YZRB, the decreasing trends were in the range of −1.29—3.02 days/decade (*p* < 0.01). The increasing trends of TXx and SU were generally higher in the YLRB than those in the YZRB during the historical period, but lower in the future period. The DTR decreased significantly during the historical period but increased significantly under the RCP4.5 and 8.5 scenarios.

The extreme precipitation indices generally increased faster in the YZRB than those in the YLRB, except the CDD, which showed significant decrease trends in the two basins, especially in the YLRB. At last, CWD generally showed significant increase trends in the two basins in the historical period and under the RCP4.5 scenario; under the RCP8.5 scenario, however, CWD decreased significantly in both the YLRB and YZRB.

## 4. Discussion

**Figure 11:**
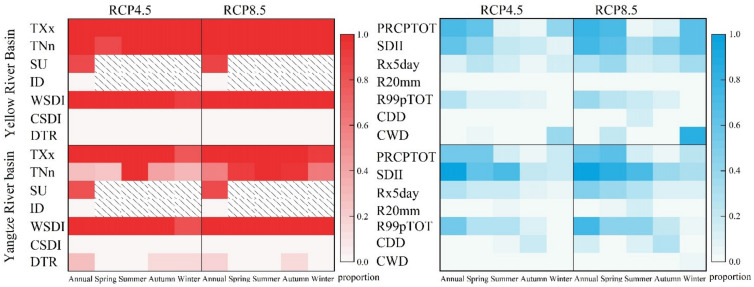
The proportion of the area with the SN value greater than one in the study areas under the RCP 4.5 and 8.5 scenarios.

Figure 11 shows the proportion of the area with SN value greater than one in the study areas under the RCP4.5 and 8.5 scenarios. The reliability of the TXx, TNn, SU, and WSDI was higher than the ID, CSDI, and DTR in the YLRB and YZRB, and the reliability under the RCP8.5 scenario was higher than that of 4.5 scenario. The extreme precipitation indices generally showed lower reliability compared with the extreme temperature in the study areas and under different scenarios [88,89].

The spatial distributions of the extreme temperature indices during the baseline period revealed notable relationships with the terrain or altitude [41]. For example, the TXx, TNn, SU, WSDI, and CSDI indices were generally low in the western regions of the two basins, which are the eastern parts of the Tibetan Plateau, where the altitude is generally higher than 3000 m, as well as on the Loess Plateau (northwestern YLRB). However, these indices were high in the Sichuan Basin and the lower reaches of the YZRB and YLRB, where the altitude is generally lower than 500 m. Most of the extreme precipitation indices also exhibited clear geographical differences. For example, extreme precipitation events generally occurred in the lower reaches of the YZRB but were rarely observed in the western regions of the two basins, which may be mainly influenced by monsoons.

**Figure 12:**
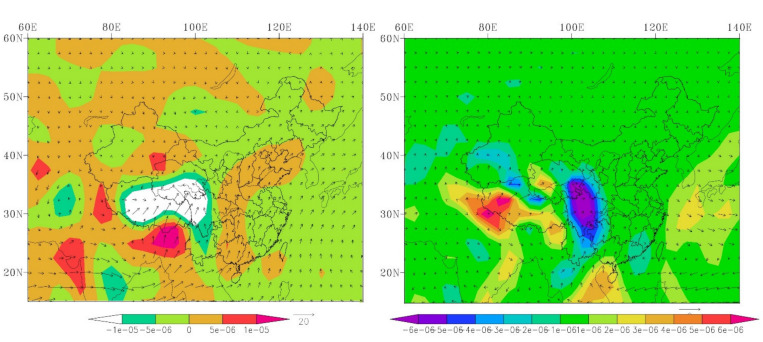
The summer and winter mean water vapor flux (vector arrow, g/cm/hPa/s) and water vapor flux divergence (shadow, g/cm^2^/hPa/s) at 850 hPa during 1971–2000.

We also calculated the average value of water vapor flux and water vapor flux divergence at 850 hPa in summer and winter from 1971–2000 (Figure 12). Along the western margin of the Sichuan Basin, water vapor transport is blocked by the Tibetan Plateau, and a humid climate was thus observed in the southwest region of the Tibetan Plateau [35]. Overall, the risk of extreme cold events is relatively high on the eastern Tibetan Plateau and the Loess Plateau regions, where the extremely high precipitation is relatively low and the area is prone to drought events. The risk of extreme hot temperatures were relatively high in the middle and lower reaches of the YZRB, where the extreme precipitation events are relatively high.

**Figure 13:**
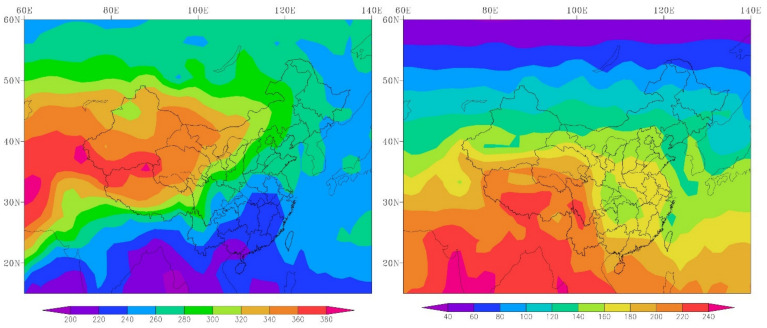
The summer and winter mean downward solar radiation flux (W/m^2^) during 1971–2000. It is not the net downward shortwave radiation flux.

In general, the warm-temperature indices in the future decade in the two basins generally exhibited increase trends in the 2020s and 2050s periods in the two basins under the RCP4.5 and RCP8.5 scenarios, but the extreme high-temperature indices in the YZRB increased faster than those in the YLRB, such as the TXx, SU, and WSDI indices. In the YLRB, the extreme low-temperature index increased faster than that in the YZRB (the TNn index), and the ID index also decreased faster than that in the YZRB, which agrees with the study of Li et al. [90]. The warm regions were generally located on the southeastern Tibetan Plateau and northern YLRB. This area may be the focus because these relatively high-altitude regions receive more positive albedo-temperature feedback, and this capacity creates a higher temperature increase than that in the relatively low-altitude regions in the YLRB and YZRB [46]. However, in the 2080s period, the warming trends in the two basins slowed, and the temperature even became colder in certain regions. Under the RCP8.5 scenario, the two basins would continue to warm in future decades, and in the Tibetan Plateau region, the TNn, SU and WSDI indices would increase faster than those in the other regions, and the ID index would decrease considerably. Figure 13 shows the summer and winter mean downward solar radiation flux during 1971–2000. The radiation value was higher in northwest of China in summer and southwestern China in winter, while the spatial distribution of surface solar radiation was not the same as that of extreme high temperatures, indicating that surface solar radiation may not be the main cause of the spatial distributions of extreme high temperature, but the study of Hu et al. [91] noted that the net surface radiation flux and 500-hPa geopotential height indicated an enhancement of the net radiation in northern China and a weakening of the East Asian trough in winter under the RCP4.5 and RCP8.5 scenarios, which may explain the increase in the winter temperature in the YLRB.

In the future, the regional mean extreme precipitation events exhibited limited increase trends in the two basins under the RCP4.5 scenario, and the increases were mainly found in the YZRB from 2020–2060. The flood peaks in the YZRB also increased 0.3–13.1% in this period [92]. Over the last decade of the 21st century, the frequency of extreme precipitation events was reduced in the middle and lower reaches of the YZRB. Under the RCP8.5 scenario, the rate of extreme precipitation events would continue to increase, especially on the eastern Tibetan Plateau and lower reaches of the YZRB. However, the number of extreme wet days would decrease, indicating that extreme precipitation would occur frequently.

The air temperature is an important factor influencing the mean precipitation and extreme precipitation. With increasing air temperature, the water vapor in the atmosphere increases nonlinearly with the temperature, which is responsible for the increase in precipitation. However, the warming climate increases the water-holding capacity of the atmosphere and thus increases the atmospheric precipitable water. A more stable atmospheric structure makes it more difficult for water vapor to condense and precipitate, but the precipitation intensity can increase once an event occurs [46]. In addition, Wu et al. [93] noted that the water vapor flux convergence under the RCP8.5 scenario exhibited large increases in the eastern and western YZRB but decreases in the central YZRB and northeastern YLRB, which would contribute to the changes in extreme precipitation events in these two basins. Rai et al. [94] reported that the mean precipitation exhibited strong and significant relationships with extreme precipitation events in the mid-late 21st century. Finally, Zhou et al. [95] also distinguished changes in the precipitation characteristics due more to external radiative forcing than to the internal climate variability.

## 5. Conclusions

In summary, the spatiotemporal distributions of seven extreme temperature indices and seven extreme precipitation indices based on 16 CMIP5 models in the YLRB and YZRB in China from 1961 to 2099 were analyzed. The statistical analysis indicated that 10 and 14 CMIP5 models met the requirements for the calculation of these extreme temperature and precipitation indices, respectively, and the REA method provided the best simulation results of the extreme climate indices for the baseline period (1971–2000); the future trends (2010–2099) of the extreme climate indices were studied based on the REA values.

The spatial distributions of the warm-temperature indices generally revealed high values in the Sichuan Basin and the middle and lower reaches of the YZRB, followed by the Loess Plateau, and low values in the Tibetan Plateau areas. The REA method usually overestimated the temperature indices in the YZRB and YLRB; for example, the ensemble TXx, TNn, SU, ID, and DTR index values usually agreed well with the observed values, except WSDI and CSDI indices, and only the ensemble TNn and WSDI index values in the YZRB were underestimated. In addition, most of the observed extreme precipitation indices over the baseline period were generally high in the middle and lower reaches of the YZRB and low on the Tibetan Plateau and Loess Plateau. The ensemble extreme precipitation indices were also overestimated by the REA method, especially in the central-eastern YZRB, but the PRCPTOT index was underestimated in the YLRB and YZRB.

Warming trends were observed in the YLRB and YZRB in the 21st century under the RCP4.5 and RCP8.5 scenarios. The warm-temperature indices (the TXx, SU and WSDI indices) in the YZRB increased faster, but the cold-temperature indices such as the TNn and ID indices changed faster than those in the YZRB. Under the RCP4.5 scenario, maximum rates of the warming trends generally occurred in the 2050s period in the YZRB, while in the 2080s period, the warming trends would slow down and even become negative in certain regions, such as in the central area of the YZRB. Under the RCP8.5 scenario, the warming trends continued, especially on the Tibetan Plateau and central area of the YZRB. The extreme precipitation events in the 21st century continued to increase in the YLRB and YZRB. Similar to the changes in the extreme temperature indices, the extreme precipitation indices increased faster in the 2020s and 2050s periods under the RCP4.5 scenario, especially in the central YZRB, the Tibetan Plateau, and the southeastern YLRB. In the 2080s period, the increase trends decelerated. Under the RCP8.5 scenario, extreme precipitation events continued to increase, especially in the last decades of the 21st century. In addition, the number of extreme wet days increased, indicating that extreme precipitation would appear more frequently.

The long-term anomalies of the extreme temperature and precipitation indices in the YZRB and YLRB indicated that the warming and wetting trends in the two basins were the same under the RCP4.5 and RCP8.5 scenarios before 2040. The trends would continue to increase after 2050 under the RCP8.5 scenario, but the warming trends would decrease after 2060 under the RCP4.5 scenario, while the wetting trends would even decrease after 2090.

The spatial and temporal distributions of the extreme temperature were controlled by the atmospheric circulation and the download solar radiation in this study. The future frequency and intensity changes of extreme temperature events are also affected by the background of global surface temperature warming, which is related to the increase in greenhouse gas emissions caused by human activities (such as the process of urbanization). The increase in the mean air temperature will cause increases in the mean precipitation and extreme precipitation, especially in the YZRB. The YZRB will experience stronger extreme precipitation processes in future decades than those experienced over the past half century, and an increased risk of floods will also occur. Extreme precipitation will appear more concentrated, along with a shorter duration. This outcome may occur because of the increased water vapor transport in summer and weakened East Asian winter monsoon (EAWM).

There are still certain problems that have not been resolved, such as the accuracy of the different individual models, a comparison to the estimation results obtained with CMIP6 model data, and the impacts of climate change on surface processes. These aspects will be examined in depth in future work.

## Figures and Tables

**Table 1:** List of 22 CMIP5 models used in this study.

No.	Model	Institute ID (Modeling Center or Group)	Resolution (Lon × Lat)	Tm, Tx, Tn	Pre
1	ACCESS1.0	CSIRO-BOM (Commonwealth Scientific and Industrial Research Organization (CSIRO) and Bureau of Meteorology (BOM), Australia)	192 × 145	√	
2	ACCESS1.3			√	
3	CCSM4	NCAR (National Center for Atmospheric Research)	288 × 192	√	√
4	CMCC-CM	CMCC (Centro Euro-Mediterraneo per I Cambiamenti Climatici)	480 × 240	√	
5	CMCC-CMS		192 × 96	√	√
6	CSIRO-Mk3.6.0	CSIRO-QCCCE (Commonwealth Scientific and Industrial Research Organization in collaboration with Queensland Climate Change Centre of Excellence)	192 × 96	√	√
7	CanESM2	CCCMA (Canadian Centre for Climate Modelling and Analysis)	128 × 64		√
8	HadGEM2-AO	NIMR/KMA (National Institute of Meteorological Research/Korea Meteorological Administration)	192 × 144	√	√
9	HadGEM2-CC	MOHC (Met Office Hadley Centre)	192 × 144		√
10	HadGEM2-ES		192 × 96	√	
11	IPSL-CM5B-LR	IPSL (Institute Pierre-Simon Laplace)	96 × 96	√	√
12	IPSL-CM5A-MR		144 × 143	√	
13	MPI-ESM-LR	MPI-M (Max Planck Institute for Meteorology)	192 × 96	√	√
14	MPI-ESM-MR			√	√
15	NorESMl-M	NCC (Norwegian Climate Centre)	144 × 96	√	
16	INMCM4	INM (Institute for Numerical Mathematics)	180 × 120	√	√

**Table 2:** The average and trends of Tm, Tn, Tx, and Pre from observed and downscaled model data during 1971–2000.

	The Yellow River Basin							
	Average				Trend (Unit/Decade)			
	Pre (mm)	Tm (°C)	Tn (°C)	Tx (°C)	Pre	Tm	Tn	Tx
OBS	449.18	8.49	2.22	14.75	−12.38	0.34 **	0.33 **	0.36 *
ACCESS1.0		6.06	1.22	10.79		0.26 **	0.19	0.33 **
ACCESS1.3		6.44	2.18	10.67		0.23 *	0.32 **	0.15 **
CCSM4	722.75	5.31	−0.34	10.98	−5.01	0.04	0.04	0.03
CMCC-CM		5.26	−1.09	11.23		0.36 **	0.33 *	0.39 **
CMCC-CMS	515.1	5.94	−0.33	11.9	0.94	0.10	0.12	0.08
CSIRO-Mk3-6-0	614.84	5.98	0.42	11.77	15.24	0.13	0.14	0.13
CanESM2	1124.85				−32.51			
HadGEM2-AO	581.13	5.28	−0.23	10.47	−17.00	0.29 *	0.41 **	0.19
HadGEM2-CC	583.54				−26.45			
HadGEM2-ES		5.66	0	11.07		0.11	0.18	0.05
INMCM4	602.78	2.76	−3.04	8.55	−33.07	0.32 **	0.24	0.39 **
IPSL-CM5A-MR		7.65	2.21	13.31		0.08	0.09	0.06
IPSL-CM5B-LR	1059.59	7.19	1.5	13.11	−30.61	0.24	0.22	0.25 *
MPI-ESM-LR	617.75	7.71	2.55	12.91	8.21	0.12	0.18	0.06
MPI-ESM-MR	809.74	7.13	2.26	12.03	−13.64	0.35 **	0.36 **	0.36 **
NorESM1-M		6.6	5.56	7.65		0.08	0.09	0.06
	The Yangtze River Basin							
OBS	1192.65	15.47	11.06	19.88	26.14	0.19 *	0.23 **	0.14
ACCESS1.0		11.82	8.04	15.59		0.16	0.15	0.17
ACCESS1.3		12.46	9.55	15.37		0.27 **	0.3	0.24 *
CCSM4	1108.59	12.41	7.41	17.4	−9.93	0.02	0.08	−0.04
CMCC-CM					−25.38	0.25 **	0.26 **	0.25 **
CMCC-CMS	1320.1	12.4	7.95	16.77	−2.41	0.03	0.09	−0.04
CSIRO-Mk3-6-0	954.07	14.18	8.22	20.22	−9.32	0.15	0.19	0.1
CanESM2	1643.8				−18.55			
HadGEM2-AO	1341.86	11.48	6.94	15.98	−37.78	0.09	0.16	0.03
HadGEM2-CC	1321.37				−62.86 **			
HadGEM2-ES		11.15	7.01	15.25		0.03	0.07	0
INMCM4	1164.5	11.37	6.98	15.75	25.96	0.09	0.13	0.05
IPSL-CM5A-MR		14.13	10.49	17.79		0.04	0.04	0.05
IPSL-CM5B-LR	1916.59	13.78	9.74	17.82	8.39	0.15	0.14	0.14
MPI-ESM-LR	1375.5	12.98	9.13	16.85	−26.15	0.14	0.16	0.12
MPI-ESM-MR	1418.86	12.43	8.96	15.92	−35.53	0.27*	0.25	0.29 *
NorESM1-M		12.11	11.39	12.83		0.09	0.10	0.08

* Significant at the 95% confidence level, ** significant at the 99% confidence level (MK test).

**Table 3:** Information of 14 ETCCDI extreme climate indices.

Index	Descriptive Name	Definitions	Units
Extreme temperature indices			
DTR	Diurnal temperature range	Mean difference between TX and TN	°C
TXx	Max Tm	Maximum value of daily maximum temp	°C
TNn	Min Tn	Minimum value of daily minimum temp	°C
SU	Summer days	Count when TX (daily maximum) > 25 °C	Days
ID	Ice days	Count when TX (daily maximum) < 0 °C	Days
WSDI	Warm spell duration index	Count of days with at least 6 consecutive days when TX > 90th percentile	Days
CSDI	Cold spell duration index	Count of days with at least 6 consecutive days when TN < 10th percentile	Days
Extreme precipitation indices			
Rx5day	Highest 5-day precipitation	Maximum consecutive 5-day precipitation	mm
R99pTOT	Extremely wet day precipitation	Precipitation due to very wet days when the PR > 99th percentile of 1971–2000 daily rainfall	mm
R20mm	Heavy precipitation days	Count of days when PR ≥ 20 mm	Days
PRCPTOT	Total wet-day precipitation	Total precipitation in wet days (PR ≥ 1 mm)	mm
SDII	Precipitation intensity	Total precipitation in wet days divided by the count of the wet days	mm/day
CDD	Consecutive dry days	Maximum number of consecutive dry days with PR < 1 mm	Days
CWD	Consecutive wet days	Maximum number of consecutive wet days with PR ≥ 1 mm	Days

**Table 4:** Regional mean values of observed extreme climate indices (OSB) and differences of REA and OBS values (REA-OBS) in the Yellow River Basin and Yangtze River Basin.

Index	The Yellow River Basin		The Yangtze River Basin	
	Regional Mean OBS	Regional Mean REA	Regional Mean OBS	Regional Mean REA
TXx (°C)	29.84	30.3	30.63	30.78
TNn (°C)	−23.28	−22.59	−11.62	−11.77
SU (days)	58.28	59.43	85.46	88.02
ID (days)	60.01	60.49	24.41	24.51
WSDI (days)	11.3	12.63	15.69	13.93
CSDI (days)	15.42	18.62	15.02	17.5
DTR (°C)	12.71	12.71	10.06	10.08
PRCPTOT (mm)	441.24	438.41	963.14	959.81
Rx5day (mm)	62.36	68.01	104.35	112.78
R20mm (days)	3.37	3.46	10.99	11.4
R99pTOT (mm)	30.05	31.58	70.14	71.91
CDD (days)	55.76	59.15	36.92	39.92
CWD (days)	7.51	8.14	11.52	12.35
SDII (mm/days)	5.85	5.9	7.78	7.85

**Table 5:** The trends of extreme climate indices (unit/decade) in the Yellow River basin and Yangtze River basin for the historical period and under the RCP4.5 and 8.5 scenarios in the future period.

Index	The Yellow River Basin			The Yangtze River Basin		
	Historical	RCP4.5	RCP8.5	Historical	RCP4.5	RCP8.5
TXx	0.23 **	0.28 **	0.69 **	0.21 **	0.33 **	0.74 **
TNn	0.30 **	0.32 **	0.71 **	0.28 **	0.28 **	0.64 **
SU	1.73 **	2.22 **	4.52 **	1.68 **	2.53 **	4.95 **
ID	−2.41 **	−2.32 **	−4.59 **	−1.29 **	−1.47 **	−3.02 **
WSDI	2.08 **	4.12 **	9.04 **	2.33 **	4.66 **	9.75 **
CSDI	−2.32 **	−1.31 **	−1.55 **	−2.1 **	−1.24 **	−1.47 **
DTR	−0.04 **	0.003 **	0.004 **	−0.02 **	0.03 **	0.04 **
PRCPTOT	6.75 **	9.23 **	11.98 **	6.04 **	14.61 **	18.99 **
Rx5day	1.07 **	1.07 **	2.08 **	1.99 **	1.91 **	3.3 **
R20mm	0.08 **	0.11 **	0.17 **	0.14 **	0.24 **	0.36 **
R99pTOT	2.72 **	2.79 **	5.48 **	4.66 **	5.91 **	11.28 **
CDD	−0.20 *	−0.83 **	−1.11 **	0.23	−0.37 **	−0.37 **
CWD	0.03 **	0.06 **	−0.003	0.03 **	0.07 **	−0.01
SDII	0.06 **	0.07 **	0.11 **	0.08 **	0.1 **	0.17 **

* Significant at the 95% confidence level, ** significant at the 99% confidence level (MK test).

## Data Availability

The daily meteorological data is provided by the National Meteorological Information Center (NMIC), China Meteorological Administration (CMA) (http://data.cma.cn, accessed on 24/9/2019). The CMIP5 model data is provided by the World Climate Research Programme (CWRP), Earth System Grid Federation (ESGF, https://esgf-node.llnl.gov/projects/cmip5/, accessed on 10/2/2020).
